# Similar immunogenicity profiles between the proposed biosimilar MYL-1501D and reference insulin glargine in patients with diabetes mellitus: the phase 3 INSTRIDE 1 and INSTRIDE 2 studies

**DOI:** 10.1186/s12902-021-00797-4

**Published:** 2021-06-26

**Authors:** Bin Sun, Nilanjan Sengupta, Anita Rao, Charles Donnelly, Vinit Waichale, Arnab Sinha Roy, Shilpa Ramaswamy, Divya Pathak, Ronald R. Bowsher, Yaron Raiter, Patrick Aubonnet, Abhijit Barve

**Affiliations:** 1 Viatris Inc, 1000 Mylan Boulevard, Canonsburg, PA 15317 USA; 2 Biocon Research Limited, 20th KM, Hosur Road, Electronic City, 560100 Bangalore, India; 3 Agilex Biolabs, SA Thebarton, Australia; 4 Cliantha Research Limited, Ahmedabad, India; 5 Altasciences, QC Laval, Canada; 6 B2S Life Sciences, 97 East Monroe Street, Franklin, IN 46131 USA; 7 Viatris Inc, Turmstrasse 24, Tower 4, 6312 Steinhausen, Switzerland

**Keywords:** Biosimilar, Diabetes mellitus, Immunogenicity, Insulin glargine, MYL-1501D

## Abstract

**Background:**

MYL-1501D is a proposed biosimilar to insulin glargine. The noninferiority of MYL-1501D was demonstrated in patients with type 1 diabetes and type 2 diabetes in 2 phase 3 trials. Immunogenicity of MYL-1501D and reference insulin glargine was examined in both studies.

**Methods:**

INSTRIDE 1 and INSTRIDE 2 were multicenter, open-label, randomized, parallel-group studies. In INSTRIDE 1, patients with type 1 diabetes received MYL-1501D or insulin glargine over a 52-week period. In INSTRIDE 2, patients with type 2 diabetes treated with oral antidiabetic drugs, insulin naive or not, received MYL-1501D or reference insulin glargine over a 24-week period. Incidence rates and change from baseline in relative levels of antidrug antibodies (ADA) and anti–host cell protein (anti-HCP) antibodies in both treatment groups were determined by a radioimmunoprecipitation assay and a bridging immunoassay, respectively. Results were analyzed using a mixed-effects model (INSTRIDE 1) or a nonparametric Wilcoxon rank sum test (INSTRIDE 2).

**Results:**

Total enrollment was 558 patients in INSTRIDE 1 and 560 patients in INSTRIDE 2. The incidence of total and cross-reactive ADA was comparable between treatment groups in INSTRIDE 1 and INSTRIDE 2 (*P* > 0.05 for both). A similar proportion of patients had anti-HCP antibodies in both treatment groups in INSTRIDE 1 at week 52 (MYL-1501D, 93.9 %; reference insulin glargine, 89.6 %; *P* = 0.213) and in INSTRIDE 2 at week 24 (MYL-1501D, 87.3 %; reference insulin glargine, 86.9 %; *P* > 0.999).

**Conclusions:**

In INSTRIDE 1 and INSTRIDE 2, similar immunogenicity profiles were observed for MYL-1501D and reference insulin glargine in patients with type 1 diabetes and type 2 diabetes, respectively.

**Trial registration:**

ClinicalTrials.gov, INSTRIDE 1 (NCT02227862; date of registration, August 28, 2014); INSTRIDE 2 (NCT02227875; date of registration, August 28, 2014).

**Supplementary Information:**

The online version contains supplementary material available at 10.1186/s12902-021-00797-4.

## Background

Diabetes mellitus is characterized by chronic hyperglycemia and can be classified into several distinct categories based on the underlying mechanism of the disease [1]. In type 1 diabetes, autoimmune destruction of pancreatic beta cells leads to insulin deficiency and hyperglycemia [1, 2]. Type 2 diabetes is characterized by insulin deficiency and resistance to the physiological effects of insulin, resulting in hyperglycemia [1, 3]. While the pathophysiology is distinct, the primary goal of treatment for both type 1 and type 2 diabetes is to achieve glycemic control and reduce hyperglycemia [4].

In addition to adherence to a healthy lifestyle, exogenous insulin therapy is a typical component of managing hyperglycemia in type 1 and type 2 diabetes [2, 5]. Insulin therapy can include prandial and/or basal insulin. Prandial insulin is administered around mealtime and is a component of therapy for most patients with type 1 diabetes. Basal insulin is administered daily as a common part of the treatment regimens for patients with type 1 or type 2 diabetes. In patients with type 2 diabetes, insulin regimens are normally administered in combination with oral antidiabetic drugs (OADs) [5]. Insulin glargine is a long-acting human insulin analogue that allows for once-daily basal use in patients with type 1 or type 2 diabetes [6]. MYL-1501D (developed jointly by Viatris Inc, Canonsburg, PA, and Biocon Limited, Bangalore, India), a proposed biosimilar/follow-on biologic to insulin glargine, has an amino acid sequence identical to reference insulin glargine (Lantus®; Sanofi-Aventis US LLC, Bridgewater, NJ) [7]. In March 2020, insulins were transitioned to a new regulatory pathway in the United States: insulins are now regulated as biologics and can serve as reference products for biosimilar or interchangeable products [8].

Minor structural differences between biosimilars and reference products can potentially impact immunogenicity and result in altered safety and efficacy [9]. Immunogenic response to biologics may lead to therapeutic neutralization and hypersensitivity reactions [9, 10]. Immunogenicity of MYL-1501D was assessed in the phase 3 noninferiority studies INSTRIDE 1 (NCT02227862) and INSTRIDE 2 (NCT02227875), which compared the safety and efficacy of MYL-1501D and reference insulin glargine in patients with type 1 and type 2 diabetes, respectively. In both studies, the primary objective was demonstrated via the noninferiority of MYL-1501D to reference insulin glargine with regard to comparable glycated hemoglobin (HbA_1c_) profiles [11–13]. MYL-1501D was also found to be generally safe and well tolerated in comparison with reference insulin glargine, with a similar rate of hypoglycemic events observed [11–13]. The objective of this analysis was to determine the immunogenicity profiles in patients with type 1 diabetes and in patients with type 2 diabetes treated with MYL-1501D or reference insulin glargine.

## Methods

### INSTRIDE 1 study design

**Fig. 1:**
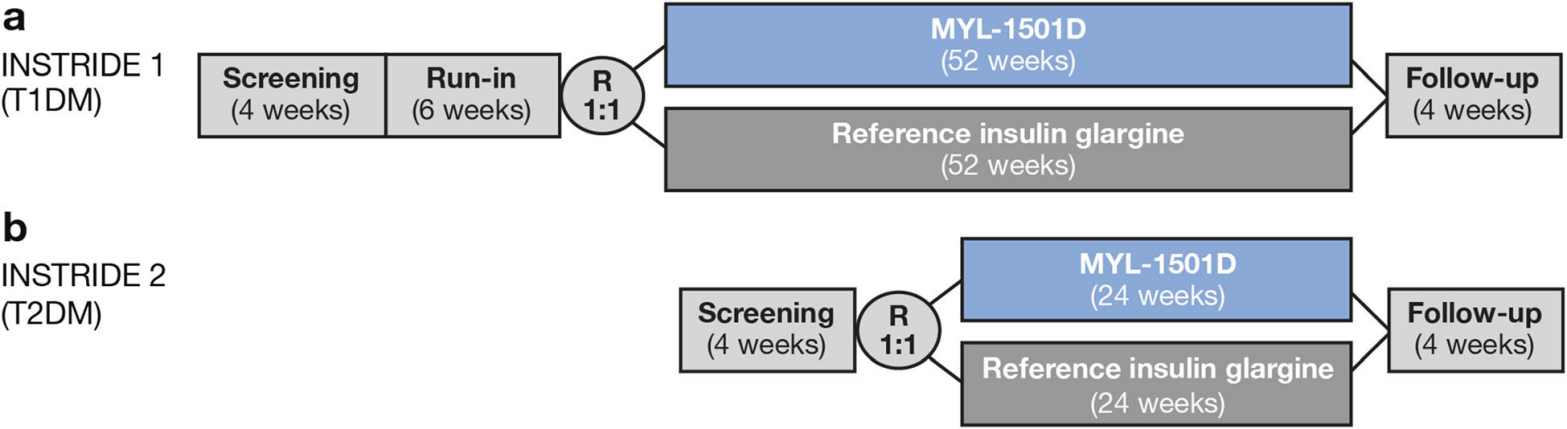
Study design of **(a)** INSTRIDE 1 and **(b)** INSTRIDE 2. T1DM, type 1 diabetes mellitus; T2DM, type 2 diabetes mellitus

INSTRIDE 1 was a multicenter, open-label, randomized, parallel-group, phase 3 study in patients with type 1 diabetes that compared the efficacy and safety of MYL-1501D with reference insulin glargine (Fig. 1a). Patients were eligible for inclusion if they had an established diagnosis of type 1 diabetes (per American Diabetes Association 2014 criteria [14]), were treated with once-daily reference insulin glargine for ≥ 3 months, had an HbA_1c_ ≤ 80 mmol/mol (9.5 %) at screening, were aged 18 to 65 years, had a fasting plasma C-peptide < 0.3 nmol/L at screening, had a stable weight for 3 months, and had a body mass index (BMI) between 18.5 and 35.0 kg/m^2^ at screening.

The maximum study duration was 66 weeks, which included a screening period of up to 4 weeks followed by a 6-week run-in period, a 52-week treatment period, and a 4-week follow-up period. After screening, patients began the run-in period and were titrated with reference insulin glargine and insulin lispro, as needed (both administered via disposable pens), to ensure good diabetes control as determined by the investigator. Reference insulin glargine was administered via subcutaneous injection at a dose adapted to patients’ blood glucose levels; dosing of MYL-1501D was guided by self-monitored blood glucose assessments, as suggested by the reference insulin glargine dosing algorithm. Titration was performed first for reference insulin glargine, then for insulin lispro. The dosage of reference insulin glargine was investigator driven and adjusted so that patients attained a fasting preprandial blood glucose of 70 to 130 mg/dL (3.9–7.2 mmol/L); the dosage of insulin lispro was adjusted so that patients attained a targeted postprandial blood glucose of < 180 mg/dL (10.0 mmol/L). Patients were randomized 1:1 after the run-in period to continue treatment with reference insulin glargine or to receive MYL-1501D during the 52-week treatment period. All patients resumed standard-of-care treatment after 52 weeks, and a follow-up visit was scheduled at week 56.

### INSTRIDE 2 study design

INSTRIDE 2 was a multicenter, open-label, randomized, parallel-group, phase 3 study that compared the efficacy and safety of MYL-1501D with reference insulin glargine in patients with type 2 diabetes treated with OADs, insulin naive or not (Fig. 1b). Patients were eligible for inclusion in the study if they had an established diagnosis of type 2 diabetes (per American Diabetes Association 2014 criteria [14]) for ≥ 1 year before screening, were on a stable dose of an OAD for ≥ 3 months before screening, were aged 18 to 65 years, and had undergone C-peptide testing if latent autoimmune diabetes was suspected. Insulin-naive patients (those never prescribed insulin or an insulin analogue on a regular basis) were required to have HbA_1c_ > 58 mmol/mol (7.5 %) to ≤ 91 mmol/mol (10.5 %). Patients receiving once-daily insulin glargine were required to be on a stable dose for ≥ 3 months before screening and to have HbA_1c_ < 91 mmol/mol (10.5 %).

The maximum study duration was 32 weeks, which included a screening period of up to 4 weeks followed by a 24-week treatment period and a 4-week follow-up period. After the 4-week screening period, patients were randomized 1:1 to MYL-1501D or reference insulin glargine. During the initial 12 weeks of the treatment period, MYL-1501D and reference insulin glargine dose was titrated without modifying the dose of OADs. The recommended initial dose of MYL-1501D and reference insulin glargine for insulin-naive patients was 10 units (or 0.2 U/kg) once daily, adjusted weekly as required, in accordance with the reference insulin glargine algorithm. Doses were adjusted so that patients attained a fasting preprandial self-monitored blood glucose of 70 to 130 mg/dL (3.9–7.2 mmol/L). During weeks 12 to 24 of treatment, MYL-1501D and reference insulin glargine dosage was maintained with minimal titration and the OAD dose (other than rescue medications) remained unchanged.

 Both INSTRIDE 1 and INSTRIDE 2 were conducted in accordance with the general principles set forth in the International Ethical Guidelines for Biomedical Research Involving Human Subjects, the International Council for Harmonization Guidelines for Good Clinical Practice, and the Declaration of Helsinki. The protocols were reviewed and approved by independent ethics committees (ECs)/institutional review boards (IRBs) in accordance with local legal regulations. The central IRB for INSTRIDE 1 and INSTRIDE 2 was Quorum Review IRB (Seattle, WA). Central ECs for INSTRIDE 1 were Eticka komise Fakultni nemocnice Kralovske Vinohrady (Praha, Czech Republic), Tallinn Medical Research Ethics Committee (Tallinn, Estonia), Sächsische Landesärztekammer Ethikkommission (Dresden, Germany), Medical Research Council Ethics Committee for Clinical Pharmacology (Budapest, Hungary), Ethics Committee for Clinical Trials of Medicinal Products (Riga, Latvia), Comisia Naţională de Bioetică a Medicamentului şi a Dispozitivelor Medicale (Bucharest, Romania), Eticka Komisia FNsP F.D. Roosevelta Namestie L. Svobodu (Banska Bystrica, Slovak Republic), University Hospital of Leicester NHS Trust (Leicestershire, UK) and for INSTRIDE 2 was Eticka Komisia FNsP F.D. Roosevelta Namestie L. Svobodu (Banska Bystrica, Slovak Republic; see Additional file 1 for a list of all local ECs, as well as the central IRB and ECs). All patients provided written informed consent before enrollment in the study.

### Immunogenicity assays

Immunogenicity analyses were conducted in the safety population, which consisted of all patients from INSTRIDE 1 and INSTRIDE 2 who had received at least 1 dose of study drug. In both studies, blood samples for immunogenicity assessment were collected at screening; baseline; and weeks 2, 4, 12, and 24, while in the INSTRIDE 1 study, samples were also collected at weeks 36 and 52.

Two conventional radioimmunoprecipitation assays (RIPA) were employed for the assessment of antidrug antibodies (ADA). A 2-assay approach, applied in a blinded fashion, was used because of the potential structural differences between drug products arising from the different host cells used in production. The 2 assays were identical except for the use of a unique tracer: ^125^I-Lantus, designated as the ‘IGlar assay,’ and ^125^I-MYL-1501D, designated as the ‘MYL-1501D assay.’ In both assays, samples underwent a pre-treatment step that included acid dissociation to release any anti-insulin antibodies complexed with free drug, followed by charcoal adsorption of the free insulin analogue. The treated samples were then incubated with a fixed amount of each tracer under the following conditions: assay buffer only (no inhibitor), assay buffer containing excess unlabeled MYL-1501D, assay buffer containing excess unlabeled insulin glargine, and assay buffer containing excess unlabeled human insulin.

Antidrug antibody complex formation with the tracers was measured via gamma counting and expressed as a percentage of bound to total radioactivity (%B/T).

Radioimmunoprecipitation assays for the detection and characterization of ADA employed a multitiered testing strategy that consisted of screening (no inhibition), confirmation (competitive inhibition with excess MYL-1501D and reference insulin glargine), and characterization (competitive inhibition with excess human insulin). A blended approach was used for the sample analysis, in which screening, confirmatory, and characterization testing were performed within the same assay run. Total ADA and insulin cross-reactive ADA were reported in terms of percentage specific binding (%SB), which is the difference between the percentage of bound to total radioactivity (%B/T) for the uninhibited and inhibited samples for each assay (Table 1). Analogous to titer values, the %SB is the relative amount of antibody present in the samples after subtraction of the response due to non-specific binding. Cut points for these assessments were determined based on %SB during assay validation and applied during sample analysis for scoring total and cross-reactive ADA-positive samples. For the MYL-1501D assay, cut points for total and cross-reactive ADA were 1.15 %SB and 1.05 %SB, respectively. For the IGlar assay, cut points for total and cross-reactive ADA were 1.00 %SB and 1.06 %SB, respectively. Comparisons of the relative amounts of total ADA or cross-reactive ADA present in the samples were based on the %SB value.

**Table 1:** Definitions of Percent Specific Binding Used in Radioimmunoprecipitation Assays

Assay		Percent specific binding definition
MYL-1501D	Total ADA	%B/T (no inhibitor) − %B/T (excess MYL-1501D)
	Insulin cross-reactive ADA	%B/T (no inhibitor) − %B/T (excess insulin)
	Drug-specific ADA	%B/T (excess IGlar) − %B/T (excess MYL-1501D)
IGlar	Total ADA	%B/T (no inhibitor) − %B/T (excess reference insulin glargine)
	Insulin cross-reactive ADA	%B/T (no inhibitor) − %B/T (excess insulin)
	Drug-specific ADA	%B/T (excess MYL-1501D) − %B/T (excess IGlar)

*ADA* antidrug antibodies, *%B/T* percentage of bound to total reactivity, *IGlar* insulin glargine

In addition, drug-specific ADA was assessed by determining the %B/T difference between samples inhibited with excess reference insulin glargine and MYL-1501D in each assay (Table 1). For this assessment, no cut point was determined or applied, and the results are reported as %B/T.

Antibodies directed against host cell proteins (HCPs) were detected with a bridging immunoassay with electrochemiluminescence (ECL) detection, which was designed and validated with a screening tier (no inhibition) and a confirmatory tier (competitive inhibition with excess HCP). Relative anti-HCP antibody levels over time were estimated using the ECL response in the screening assay.

### Incidence of local and systemic allergic reactions

Incidence of treatment-emergent local and systemic allergic reactions was summarized by treatment group within each trial.

### Statistics

In INSTRIDE 1, the immunogenicity profiles were analyzed using a mixed-effects model with repeated measurement method, which included terms for treatment, visit, treatment-by-visit, and region as fixed effects, with the baseline value as a covariate. In INSTRIDE 2, antibody percent binding profiles were analyzed using a nonparametric Wilcoxon rank sum test due to non-normal distribution of the data. Similar statistical methods of mixed-effects models were also used for mean actual ECL ratio analyses in both studies. For categorical data analyses, Fisher exact or chi-square tests were performed. All tests of treatment effects were conducted at a 2-sided alpha level of 0.05.

## Results

### Patient demographics

In INSTRIDE 1, 832 patients were screened and 558 were randomized (MYL-1501D, *n* = 280; reference insulin glargine, *n* = 278). Baseline patient characteristics were generally similar between groups (Table 2). Mean age of patients in the MYL-1501D and reference insulin glargine groups was 42.0 and 42.2 years, respectively. The majority of patients were male (MYL-1501D, 58.6 %; reference insulin glargine, 61.9 %) and most were white (MYL-1501D, 93.9 %; reference insulin glargine, 95.3 %). Mean BMI was 26.4 kg/m^2^ in the MYL-1501D group and 26.6 kg/m^2^ in the reference insulin glargine group.

**Table 2:** Baseline Patient Demographics (Randomized Populations)

	INSTRIDE 1 (T1DM)		INSTRIDE 2 (T2DM)^**a**^	
	MYL‑1501D (***n***=280)	Reference insulin glargine (***n***=278)	MYL‑1501D (***n***=277)	Reference insulin glargine (***n***=283)
Age, mean (SD), y	42.0 (12.0)	42.2 (12.0)	55.0 (7.9)	55.1 (7.5)
Sex, n (%)				
Male	164 (58.6)	172 (61.9)	147 (53.1)	165 (58.3)
Female	116 (41.4)	106 (38.1)	130 (46.9)	118 (41.7)
Race, n (%)				
White	263 (93.9)	265 (95.3)	147 (53.1)	148 (52.3)
Hispanic	6 (2.1)	3 (1.1)	73 (26.4)	76 (26.9)
Black	2 (0.7)	5 (1.8)	37 (13.4)	18 (6.4)
Asian	2 (0.7)	2 (0.7)	9 (3.2)	19 (6.7)
Hawaiian native	0 (0.0)	0 (0.0)	0 (0.0)	4 (1.4)
American Indian	0 (0.0)	0 (0.0)	0 (0.0)	1 (0.4)
Other	7 (2.5)	3 (1.1)	11 (4.0)	17 (6.0)
Geographic region, n (%)				
North America	126 (45.0)	126 (45.3)	225 (81.2)	228 (80.6)
Europe	145 (51.8)	145 (52.2)	34 (12.3)	33 (11.7)
Middle East and Africa	9 (3.2)	7 (2.5)	14 (5.1)	18 (6.4)
East Asia	0 (0.0)	0 (0.0)	4 (1.4)	4 (1.4)
BMI, mean (SD), kg/m^2^	26.4 (3.7)	26.6 (4.2)	31.6 (4.8)	31.5 (4.4)
Duration of diabetes, mean (SD), y	18.7 (11.8)	19.7 (11.3)	12.0 (7.1)	11.3 (6.0)
HbA_1c_, mean (SD), mmol/mol	57.1 (9.5)	57.2 (9.2)	65.5 (12.5)	65.3 (12.4)
HbA_1c_, mean (SD), %	7.4 (0.9)	7.4 (0.8)	8.1 (1.1)	8.1 (1.1)
FPG, mean (SD), mmol/L	9.3 (3.8)	9.1 (3.4)	8.6 (3.0)	8.6 (3.1)
Mealtime insulin dose, mean (SD), U/kg	0.36 (0.17)	0.35 (0.15)	—	—
Daily basal insulin dose, mean (SD), U/kg^b^	0.31 (0.12)	0.32 (0.15)	0.22 (0.25)	0.24 (0.28)
Total daily insulin dose, mean (SD), U/kg	0.68 (0.23)	0.69 (0.24)	—	—

*BMI* body mass index, *FPG* fasting plasma glucose, *HbA*_1c_ glycated hemoglobin, *T1DM* type 1 diabetes mellitus, *T2DM* type 2 diabetes mellitus

^a^One patient in each treatment group did not receive study drug during the treatment period

^b^Only non–insulin-naive patients were included in the insulin dose summary for INSTRIDE 2

In INSTRIDE 2, of 951 patients screened, 560 were randomized 1:1 to MYL-1501D and reference insulin glargine; 228 patients were insulin naive. Mean age was 55.0 years in the MYL-1501D group and 55.1 years in the reference insulin glargine group (Table 2). The majority of patients were male (MYL-1501D, 53.1 %; reference insulin glargine, 58.3 %) and white (MYL-1501D, 53.1 %; reference insulin glargine, 52.3 %). Mean BMI was 31.6 kg/m^2^ in the MYL-1501D group and 31.5 kg/m^2^ in the reference insulin glargine group.

### Immunogenicity

#### Changes in ADA percent binding

**Fig. 2:**
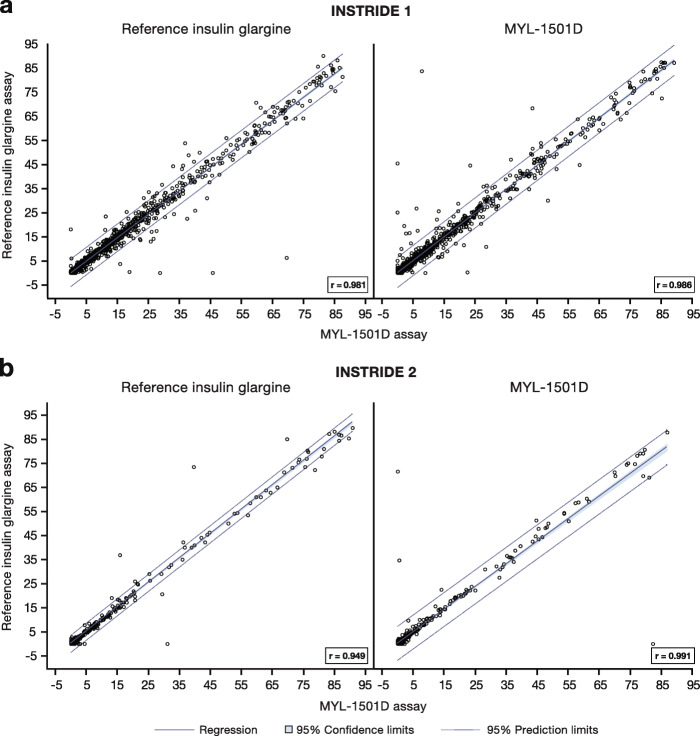
Fit plot of cross-reactive insulin antibody percent specific binding assays in **(a)** INSTRIDE 1 and **(b)** INSTRIDE 2

**Fig. 3:**
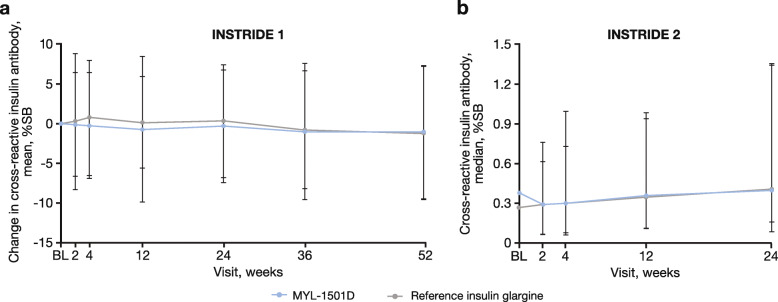
**a** Mean change in cross-reactive ADA by visit using the MYL-1501D assay in INSTRIDE 1^a^ and **b** actual median cross-reactive ADA by visit using the MYL-1501D assay in INSTRIDE 2.^b^ ADA, antidrug antibodies; BL, baseline; %SB, percentage specific binding. ^a^Error bars represent the SD. ^b^Error bars represent the interquartile range

In INSTRIDE 1, the MYL-1501D and reference insulin glargine assays were determined to be highly correlative (Fig. 2a), which suggested comparable ability of the 2 assays to detect ADA from either drug. These findings indicate that antibodies against either drug may be evaluated using the same assay. Mean changes from baseline total or cross-reactive ADA percent binding profiles were similar in the MYL-1501D and reference insulin glargine groups using the MYL-1501D assay over the 52-week treatment period (Fig. 3a). No statistically significant changes from baseline over time in total ADA-positive response within each treatment group were observed during the study, except for 1 time point. There was a statistically significant change from baseline in total ADA-positive response at week 52 for the reference insulin glargine group (mean change [SD], -1.233 [8.6230]; *P* = 0.027) but not for the MYL-1501D group (mean change [SD], -0.892 [8.5497]; *P* = 0.103). There were no statistically significant changes from baseline in total or cross-reactive ADA percent binding between treatment groups at any time point using the MYL-1501D and reference insulin glargine assays.

In INSTRIDE 2, the MYL-1501D and reference insulin glargine assays were also highly correlative (Fig. 2b). Actual median total or cross-reactive ADA percent binding profiles were similar over the 24-week treatment period (Fig. 3b). There were no statistically significant changes from baseline in total or cross-reactive ADA percent binding between treatment groups at any time point using the MYL-1501D or reference insulin glargine assays. No statistically significant changes from baseline were observed in total or cross-reactive ADA percent binding between treatment groups in the insulin-naive subgroups at any time point for each insulin assay.

#### ADA-positive responses

**Fig. 4:**
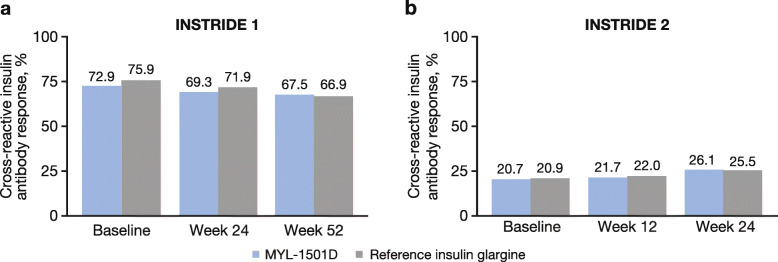
Proportion of patients with a cross-reactive insulin antibody response using the MYL-1501D assay in **(a)** INSTRIDE 1 and **(b)** INSTRIDE 2

In INSTRIDE 1, the proportion of patients who met the criteria for a total ADA-positive response (%SB ≥ 1.15 %) or cross-reactive ADA-positive response (%SB ≥ 1.05 %) was similar between the treatment groups at all time points, using the MYL-1501D assay (*P* > 0.05; Fig. 4a). At baseline, 72.9 and 75.9 % of patients had a cross-reactive ADA-positive response in the MYL-1501D and reference insulin glargine groups, respectively; at week 52, 67.5 and 66.9 %, respectively, had a cross-reactive ADA-positive response. Similarly, using the reference insulin glargine assay, the proportions of patients who met the criteria for a total or cross-reactive ADA-positive response were similar between the treatment groups at all time points (data not shown; *P* > 0.05).

In INSTRIDE 2, the proportion of patients with total or cross-reactive ADA-positive response was also similar between groups at all time points, using the MYL-1501D assay (*P* > 0.05; Fig. 4b). At baseline, 20.7 and 20.9 % of patients in the MYL-1501D and reference insulin glargine groups, respectively, had a cross-reactive ADA-positive response; at week 24, 26.1 and 25.5 % of patients, respectively, had a cross-reactive ADA-positive response. For the reference insulin glargine assay, a similar proportion of patients met the criteria for a total or cross-reactive ADA-positive response at all time points (data not shown; *P* > 0.05).

#### Anti-HCP antibodies

**Fig. 5:**
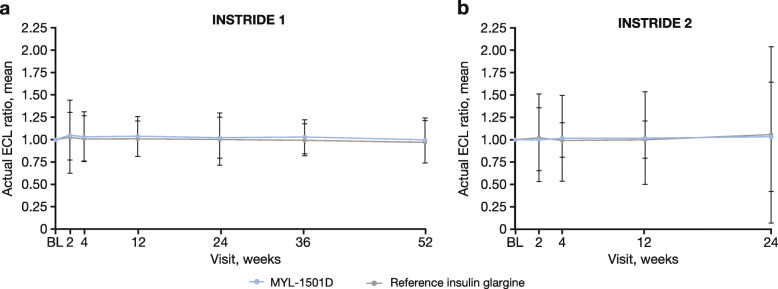
Mean actual ECL ratio (anti-HCP antibodies) in **(a)** INSTRIDE 1 and **(b)** INSTRIDE 2. Error bars represent the SD. ECL ratio is defined as ECL/ECL at baseline. BL, baseline; ECL, enhanced electrochemiluminescence; HCP, host cell protein

At baseline in INSTRIDE 1, 95.7 % of patients in the MYL-1501D group and 94.2 % of patients in the reference insulin glargine group were positive for anti-HCP antibodies. At week 52, 93.9 % of patients in the MYL-1501D group and 89.6 % of patients in the reference glargine group were positive for anti-HCP antibodies. No statistically significant differences were observed between the treatment groups at any time point in change from baseline in ECL ratio (no change in the ECL ratio was reflected by a mean of 1; Fig. 5a).

In INSTRIDE 2, 93.5 % of patients in the MYL-1501D group and 95.0 % of patients in the reference insulin glargine group tested positive for anti-HCP antibodies at baseline. At week 24, 87.3 % of patients in the MYL-1501D group and 86.9 % of patients in the reference glargine group tested positive for anti-HCP antibodies. No statistically significant differences were observed in change from baseline in ECL ratio between the treatment groups at any time point (Fig. 5b).

#### Incidence of local and systemic allergic reactions

The number of patients reporting treatment-emergent local or systemic allergic reactions and incidence of hypoglycemic events in both INSTRIDE 1 and INSTRIDE 2 are shown in Table 3. In general, incidences of local and systemic allergic reactions were low and numerically similar between treatment groups. Incidences of overall as well as severe hypoglycemic events were similar between treatment groups for both INSTRIDE 1 and INSTRIDE 2.

**Table 3:** Summary of Local and Systemic Allergic Reactions^a^ and Incidence^b^ of Hypoglycemic Events (Safety Population)

n (%)	INSTRIDE 1 (T1DM)		INSTRIDE 2 (T2DM)	
	MYL‑1501D (***n***=280)	Reference insulin glargine (***n***=278)	MYL‑1501D (***n***=276)	Reference insulin glargine (***n***=282)
Patient with reaction	5 (1.8)	6 (2.2)	4 (1.4)	2 (0.7)
Local	3 (1.1)	4 (1.4)	2 (0.7)	1 (0.4)
Systemic	2 (0.7)	2 (0.7)	2 (0.7)	1 (0.4)
Hypoglycemic incidence	273 (97.5)	269 (96.8)	130 (47.1)	136 (48.2)
Severe hypoglycemic incidence	11 (3.9)	13 (4.7)	0 (0)	1 (0.4)

*T1DM* type 1 diabetes mellitus, *T2DM* type 2 diabetes mellitus

^a^A patient was counted only once if the patient had multiple injection site reactions or signs/symptoms in the same location

^b^Incidence was defined as a patient who experienced at least 1 episode of a hypoglycemic event

## Discussion

Overall, similar immunogenicity profiles were observed for MYL-1501D and reference insulin glargine in patients with type 1 diabetes and in patients with type 2 diabetes. However, several factors prevent direct comparison of the immunogenicity profiles of patients with type 1 diabetes in INSTRIDE 1 and patients with type 2 diabetes in INSTRIDE 2. First, the studies were not powered for this comparison. Consistent with prior studies of potential insulin glargine biosimilars, INSTRIDE 1 and INSTRIDE 2 were structured to assess immunogenicity of type 1 and type 2 diabetes patient populations independently [15]. Second, there are known differences in the immune response in patients with type 1 and type 2 diabetes that may affect their respective immunogenicity profiles [16]. For example, insulin-specific antibodies have been reported to be consistently higher in patients with type 1 diabetes compared with patients with type 2 diabetes [17]. Finally, the patient populations in the 2 trials differed; relative to patients with type 1 diabetes in INSTRIDE 1, the population of patients with type 2 diabetes in INSTRIDE 2 was older, more racially diverse, and had a higher BMI. These attributes are consistent with patients with diabetes in the real world [18].

In INSTRIDE 1, similar baseline levels and changes from baseline in ADA incidence, relative antibody levels, incidence of ADA cross-reactivity to human insulin, and drug-specific ADA were observed throughout the time points of the study in patients with type 1 diabetes treated with MYL-1501D and reference insulin glargine. There was a statistically significant difference in %SB change from baseline for total ADA in the reference insulin glargine group at week 52 using the reference insulin glargine assay, although the clinical relevance of this observation is unknown. Statistical significance was not reached at any other time point, and the incidence of total ADA was not significantly different between treatment groups at any postbaseline time point. Most patients in INSTRIDE 1 were positive for total ADA, consistent with previous studies that have demonstrated a prevalence of insulin antibodies in patients with type 1 diabetes [15, 19]. Anti-HCP antibodies were consistent from baseline to the end of the study period and between the MYL-1501D and reference insulin glargine groups as shown by the exploratory analysis of ECL ratio, demonstrating an overall safe immunogenicity profile. Furthermore, the incidence of potential ADA-induced adverse events (i.e., local and systemic allergic reactions) was low and similar between treatment groups.

In INSTRIDE 2, incidence of total ADA and insulin cross-reactive antibodies at baseline and throughout the study was similar for MYL-1501D and reference insulin glargine, and most patients were negative for total ADA and insulin cross-reactive antibodies. Levels of anti-HCP antibodies, as estimated by ECL ratio, were consistent from baseline to the end of the study when comparing treatment groups. The immunogenicity profiles of MYL-1501D and reference insulin glargine were similar in insulin-naive and insulin–non-naive patients. As observed in INSTRIDE 1, incidence of local and systemic allergic reactions was low and similar between treatment groups.

One strength of these studies was the use of a multitiered sample analysis for immunogenicity testing. Application of a validated radioimmunoprecipitation assay design for detection of anti-insulin antibodies is consistent with previously published immunogenicity assessments of other insulin biosimilars [15, 20]. Multitiered testing is the US Food and Drug Administration–recommended approach because of differing clinical trial sizes and the necessity of testing patient samples at a variety of time points [21]. Additionally, titrations during both INSTRIDE 1 and INSTRIDE 2 were overseen by titration committees that reviewed patients’ insulin doses and self-measured glucose levels. Investigators were queried upon deviation from the titration algorithm without explanation to help avoid a bias toward one treatment over the other and ensure proper titration of dose to actual blood glucose values.

One possible limitation of INSTRIDE 1 and INSTRIDE 2 was their open-label study design. There were distinct differences in the injectable pens and packaging of MYL-1501D and reference insulin glargine, thus patient blinding was not possible. However, to minimize potential bias, the treatment assignments were not revealed to the central laboratory during safety (clinical safety laboratory and immunogenicity) and efficacy (HbA_1c_) analyses. Therefore, the study design of the original trials should not influence the immunogenicity results.

## Conclusions

The immunogenicity profiles of MYL-1501D and reference insulin glargine in INSTRIDE 1 and INSTRIDE 2 were similar between the treatment groups. No clinically meaningful differences were noted between the treatment groups. The clinical impact of immunogenicity observed in these trials cannot be determined since some patients in both trials had previously been exposed to insulin therapy, which may have resulted in the presence of reactive antibodies before study initiation in these patients. These data, along with the previously reported efficacy data that showed noninferiority of MYL-1501D, demonstrate the similarity of MYL-1501D to reference insulin glargine in patients with type 1 diabetes and patients with type 2 diabetes and support the use of MYL-1501D as a basal insulin analogue for the treatment of diabetes.

## Supplementary Information

**Additional file 1.** List of IRBs and ECs

## Data Availability

The data sets generated during and/or analyzed during the current study are available from the corresponding author on reasonable request.

## Abbreviations

ADA: Antidrug antibodies
BMI: Body mass index
%B/T: Percentage of bound to total radioactivity
ECL: Electrochemiluminescence
HbA_1c_: Glycated hemoglobin
HCP: Host cell protein
IGlar: Insulin glargine
OAD: Oral antidiabetic drug
%SB: Percentage specific binding
