# Supplementary material for: Similar immunogenicity profiles between the proposed biosimilar MYL-1501D and reference insulin glargine in patients with diabetes mellitus: the phase 3 INSTRIDE 1 and INSTRIDE 2 studies
Source: BMC Endocr Disord. 2021 Jun 26;21:129. doi: 10.1186/s12902-021-00797-4 (PMC8235862; doi:10.1186/s12902-021-00797-4)
Supplement: Supplementary file 1 — Additional file 1. List of IRBs and ECs [file 12902_2021_797_MOESM1_ESM.pdf]

ADDITIONAL FILE 1. List of IRBs and ECs

Similar Immunogenicity Profiles Between the Proposed Biosimilar MYL-1501D and Reference Insulin Glargine in Patients With Diabetes Mellitus: The Phase 3 INSTRIDE 1 and INSTRIDE 2 Studies

Bin Sun, Nilanjan Sengupta, Anita Rao, Charles Donnelly, Vinit Waichale, Arnab Sinha Roy, Shilpa Ramaswamy, Divya Pathak, Ronald R. Bowsher, Yaron Raiter, Patrick Aubonnet, Abhijit Barve

IRBs and ECs for INSTRIDE 1

Study center number	Name of the IRB	Chairperson
Central IRB - Canada; Centers 01002, 01004, 01006, 01008	Quorum Review IRB 1501 Fourth Ave Suite 800 Seattle, Washington 98101 United States	Stephen J. Rosenfeld, MD, MBA
LEC - Canada; Center 01010	Health Research Ethics Board of Alberta - Clinical Trials Committee 1500, 10104 - 103 Avenue NW 8625 – 112 St. Edmonton, Alberta T6J 4A7 Canada	Sunil Desai, HREBA-CTC
LEC - Canada; Center 01009	The University of Manitoba, Biomedical Research Ethics Board, Bannatyne Campus 770 Bannatyne Avenue P126 Winnipeg, Manitoba R3E 3P4 Canada	Ian Maclean, PhD
LEC - Canada; Center 01007	McGill University Health Center-Research Ethics Board/Esther Boyle 1650 Cedar Avenue Room C7-118 Montreal, Quebec H3A 1A4 Canada	Dr Terry Chow
CEC - Czech Republic; Centers 02001, 02002, 02003, 02004, 02005, 02006, 02007, 02008, 02009	Eticka komise Fakultni nemocnice Kralovske Vinohrady Srobarova 50 Praha 10, 100 34 Czech Republic	Prof MUDr Jan Pachl, CSc
LEC - Czech Republic; Center 02001	Eticka komise FN Brno Jihlavska 20 Brno, 625 00 Czech Republic	PharmDr Sarka Kozakova, MBA
LEC - Czech Republic; Center 02003	Eticka komise Vitkovicke nemocnice a.s. Zaluzanske 1192/15 Ostrava-Vitkovice, 703 84, Czech Republic	MUDr Vladimir Cizek
LEC - Czech Republic; Center 02009	Eticka komise NZZ CLINTRIAL, s.r.o. Pocernika 1427/16 Praha 10, 10 000, Czech Republic	JUDr Jaroslav Hofman
LEC - Czech Republic; Center 02008	Eticka komise Fakultni nemocnice u sv. Anny v Brne Pekarska 53 Brno, 656 91 Czech Republic	Prof MUDr Vladimir Soska, CSc

Study center number	Name of the IRB	Chairperson
CEC - Estonia; Centers 03001, 03002, 03003, 03004, 03005	Tallinn Medical Research Ethics Committee<br>Hiiu 42<br>Tallinn, 11619<br>Estonia	J. Pulluste
CEC - Germany; Centers 04001, 04002, 04004, 04005, 04006, 04007, 04008, 04009, 04010, 04011	Sächsische Landesärztekammer Ethikkommission<br>Schützenhöhe 16-18<br>Dresden,Sachsen 01099<br>Germany	Prof Dr med habil Bernd Terhaag
CEC - Hungary; Centers 05001, 05002, 05003, 05005, 05006, 05007, 05008, 05009, 05010,05013, 05014	Medical Research Council Ethics Committee for Clinical Pharmacology<br>Arany J. utca 6-8<br>Budapest, 1051<br>Hungary	Prof Dr Zsuzsanna Furst
CEC - Latvia; Centers 06001, 06002, 06003, 06004, 06005, 06006, 06007, 06008	Ethics Committee for Clinical Trials of Medicinal Products<br>Aizkraukles street 21 - 113<br>Riga, LV1006<br>Latvia	Jolanta Glodsteine, Dr math

Study center number	Name of the IRB	Chairperson
CEC - Romania; Centers 07001, 07002, 07003, 07004, 07005, 07006, 07007, 07008, 07009, 07010, 07011, 07012, 07013, 07014	Comisia Naţională de Bioetică a Medicamentului şi a Dispozitivelor Medicale Sos Stefan cel Mare, nr 19-21 Bucuresti, sector 2 020125 Romania	Prof Dr Dinu Antonescu
CEC - Slovak Republic; Centers 08001, 08002, 08003, 08004, 08005, 08006, 08007, 08009, 08010, 08011, 08012, 08013, 08014, 08015, 08016, 08017, 08018, 08019, 08020, 08021, 08022	Eticka Komisia FNsP F.D. Roosevelta Namestie L. Svobodu 1 Banska Bystrica, 975 17 Slovak Republic	Mgr Katarina Cupkova
LEC - Slovak Republic; Centers 08001, 08002, 08017	Eticka komisia Nitrianskeho samospravneho kraja Stefanikova tr. 69 Nitra, 949 01 Slovak Republic	Tibor Duris, Assoc Prof, MD, PhD
LEC - Slovak Republic; Center 08022	Eticka komisia Banskobystrickeho samospravneho kraja Namestie SNP 23 Banska Bystrica, 974 01 Slovak Republic	Katarina Cupkova

Study center number	Name of the IRB	Chairperson
LEC - Slovak Republic; Centers 08014, 08016	Eticka komisia Zilinskeho samospravneho kraja<br>Ul. Komenskeho 48<br>Zilina, 011 09<br>Slovak Republic	MUDr Martin Kapasny, PhD, MPH
LEC - Slovak Republic; Centers 08003, 08005, 08006	Eticka komisia Trencianskeho samospravneho kraja<br>K dolnej stanici 7282/20A<br>Trencin, 911 01<br>Slovak Republic	Doc MUDr Jan Bielik, CSc
LEC - Slovak Republic; Centers 08007, 08021	Eticka komisia Bratislavskeho samospravneho kraja<br>Sabinovska 16<br>Bratislava, 820 05<br>Slovak Republic	MUDr. Valerian Poticny, MPH
LEC - Slovak Republic; Centers 08004, 08019	Eticka komisia Kosickeho samospravneho kraja<br>Namestie Maratonu mieru 1<br>Kosice, 042 66<br>Slovak Republic	Maria Grullingova
LEC - Slovak Republic; Centers 08011, 08013, 08018	Eticka komisia Presovskeho samospravneho kraja<br>Namestie mieru 2<br>Presov, 080 01<br>Slovak Republic	MUDr. Julius Zbynovsky, MPH
LEC - Slovak Republic; Center 08009	Eticka komisia Nemocnica sv. Michala, a.s., Cintorinska 16, 811 08 Bratislava<br>Cesta na Cerveny most 1<br>Bratislava, 811 08<br>Slovak Republic	Vladimir Majtan, MD
LEC - Slovak Republic; Center 08010	Eticka komisia Narodny endokrinologicky a diabetologicky ustav, n.o.<br>Lubochna 144<br>Lubochna, 034 91<br>Slovak Republic	Kentos Peter, MUDr, PhD
LEC - Slovak Republic; Center 08020	Eticka komisia Trnavskeho samospravneho kraja<br>Starohajska 10 P.O.BOX 128<br>Trnava, 917 01<br>Slovak Republic	Rastislav Gajdos, MD
LEC - Slovak Republic; Center 08015	Eticka komisia NsP Nove Mesto nad Vahom n.o. M. R. Stefanika 1<br>Nove Mesto nad Vahom, 915 01<br>Slovak Republic	MUDr Albin Ferenec

Study center number	Name of the IRB	Chairperson
LEC - South Africa; Centers 09001, 09002, 09003, 09004, 09005, 09006, 09008, 09009, 09010, 09011, 09012, 09013, 09014, 09015, 09016, 09017	Pharma Ethics<br>123 Amcor Road<br>Lyttelton Manor<br>Centurion<br>Pretoria, Gauteng 0157<br>South Africa	Dr CSJ Duvenage
CEC - United Kingdom; Centers 10002, 10004, 10005	University Hospital of Leicester NHS Trust Gwendolen Road Leicester<br>Leicestershire<br>LE5 4PW<br>United Kingdom	Ken Willis

Study center number	Name of the IRB	Chairperson
Central IRB - United States; Centers 11001, 11002, 11003, 11004, 11005, 11006, 11007, 11009, 11010, 11011, 11012, 11013, 11014, 11015, 11016, 11017, 11019, 11020, 11021, 11022, 11023, 11025, 11026, 11027, 11028, 11029, 11030, 11031, 11033, 11034, 11035, 11036, 11037, 11039, 11040, 11041, 11042, 11043, 11044, 11046, 11047, 11048, 11053, 11055, 11056, 11058, 11059, 11060, 11061,	Quorum Review IRB<br>1501 Fourth Ave<br>Suite 800<br>Seattle, Washington 98101<br>United States	Stephen J. Rosenfeld, MD, MBA

Study center number	Name of the IRB	Chairperson
11062, 11063, 11065, 11066, 11067, 11068, 11069, 11071, 11072, 11073, 11074, 11075, 11076, 11077, 11078, 11081, 11082, 11083, 11084, 11086, 11089, 11090, 11091, 11093, 11094, 11095, 11096, 11097, 11098, 11100		
LEC - United States; Center 11024	Mercy Medical Center - DSM<br>1111 6th Avenue<br>Des Moines, Iowa 50314<br>United States	Matthew Andres, DO
LEC - United States; Centers 11008, 11032, 11052	WIRB<br>South Hill Business and Technology Center<br>1019 39th Ave SE<br>Puyallup, Washington 98374<br>United States	R. Bert Wilkins, JD, MHA, CIP
LEC - United States; Center 11050	BRANY IRB<br>10 Union Square East - 5J Suite 210<br>Lake Success, New York 11041<br>United States	Co-chairs: Mark J. Sinnett, PharmD and Ira Leviton, MD
LEC - United States; Center 11051	SUNY Upstate Medical University IRB<br>750 E. Adams Street<br>1109 WH Syracuse, New York 13210<br>United States	Stephen Graziano, MD

Study center number	Name of the IRB	Chairperson
LEC - United States; Center 11018	Creighton University IRB<br>2500 California Plaza<br>Omaha, Nebraska 68178<br>United States	Mary Kunes-Connell, RN, PhD

IRBs and ECs for INSTRIDE 2

Study center number	Name of the IRB	Chairperson
LEC - Jordan; Site 23001	Institutional Review Board Islamic Hospital<br>Al Zobier Ebn El Awam street Amman<br>Jordan	Dr Aly Ahmed Mishal
LEC - Jordan; Site 23003	Institutional Review Board King Abdullah University Hospital<br>Jordan University of Science and Technology, P.O Box 630001<br>Irbid Jordan 22110	Professor Doctor Khaled Al-Salem
LEC - Korea, Republic of; Site 24011	Yonsei University, Gangnam Severance Hospital, Institutional Review Board<br>3F, Main building, Gangnam Severacne Hospital<br>Yonsei Universtiy Health System<br>211 Eonjuro, Gangnam-gu Seoul<br>Korea 135-720	Kyung-Yul Lee
LEC - Korea, Republic of; Site 24004	Institutional Review Board of Seoul National University Hospital<br>101, Daehak-ro, Jongno-gu Seoul<br>Korea 03080	Woo Ho Kim, MD, PhD
LEC - Korea, Republic of; Site 24005	IRB of Eulji General Hospital 68, Hangeulbiseong-ro, Nowon-gu<br>Seoul Korea<br>01830	O Hyun Kwon
LEC - Korea, Republic of; Site 24014	IRB of Ajou University Hospital 164, World cup-ro, Yeongtong-gu Suwon-si, Gyeonggi-do<br>Korea 16499	Chairperson A: Mi Son Chun; Chairperson B: Jai Sung Noh; Chairperson C: Sung-Ran Cho; Chairperson D: Jin Hyuk Choi
LEC - Slovak Republic; Sites 28001, 28002	Eticka komisia Kosickeho samospravneho kraja<br>Namestie Maratonu mieru 1<br>Kosice Slovak Republic<br>042 66	Maria Grullingova
CEC - Slovak Republic; Sites 28001, 28002, 28003, 28005, 28006, 28007, 28008, 28009, 28010, 28011, 28012, 28015,	Eticka Komisia FNsP F.D. Roosevelta Namestie L. Svobodu 1<br>Banska Bystrica Slovak Republic 975 17	Mgr. Katarina Cupkova

Study center number	Name of the IRB	Chairperson
28018, 28019,28020		
LEC - Slovak Republic; Sites 28003, 28006, 28008, 28009	Eticka komisia Nitrianskeho samospravneho kraja<br>Stefanikova tr. 69<br>Nitra Slovak Republic<br>949 01	Tibor Duris, Assoc. Prof, MD, PhD
LEC - Slovak Republic; Sites 28005, 28012,28020	Eticka komisia Presovskeho samospravneho kraja<br>Namestie mieru 2<br>Presov Slovak Republic<br>080 01	MUDr Julius Zbynovsky, MPH
LEC - Slovak Republic; Sites 28007, 28019	Eticka komisia Bratislavskeho samospravneho kraja<br>Sabinovska 16<br>Bratislava<br>Slovak Republic 820 05	MUDr Valerian Poticny, MPH
LEC - Slovak Republic; Site 28011	Eticka komisia Trencianskeho samospravneho kraja<br>K dolnej stanici 7282/20A<br>Trencin Slovak Republic<br>911 01	Doc MUDr Jan Bielik, CSc
LEC - Slovak Republic; Sites 28015, 28018	Eticka komisia Zilinskeho samospravneho kraja Ul.<br>Komenskeho 48<br>Zilina Slovak Republic<br>011 09	MUDr. Martin Kapasny, PhD, MPH
LEC - South Africa; Sites 29001, 29002, 29004, 29005, 29006, 29007, 29008, 29009, 29010, 29011, 29012, 29013, 29014,29015	Pharma Ethics 123 Amcor Road Lyttelton Manor<br>Centurion Pretoria, Gauteng South Africa 0157	Dr CSJ Duvenage
LEC - Taiwan, Republic of China; Site 30001	Cardinal Tien Hospital Institutional Review<br>Board Room 821, A Building<br>No. 362 Zhongzheng Road,<br>Xindian District<br>New Taipei City, Taiwan<br>Republic of China<br>23148	Ting-Ywan Chou
LEC - Taiwan, Republic of China; Site 30003	Research Ethics Committee National Taiwan<br>University Hospital<br>7 Chung-Shan South Road Taipei, Taiwan Republic<br>of China<br>100	Hong-Nerng Ho

Study center number	Name of the IRB	Chairperson
LEC - Taiwan, Republic of China; Site 30004	Institutional Review Board of the E-DA Hospital No 6, Yida Road<br>Jiaosu Village, Yanchao District Kaohsiung City, Taiwan Republic of China<br>82445	Dr Hsi-Hsun Lin
LEC - Taiwan, Republic of China; Site 30006	Kaohsiung Medical University Chung-Ho Memorial Hospital Institutional Review Board<br>No.100 Tzyou 1st Road Kaohsiung, Taiwan Republic of China<br>807	Li-Tzong Chen, MD, PhD
LEC - Taiwan, Republic of China; Site 30008	Research Ethics Committee China Medical University & Hospital<br>No.2 YuDe Road, Taichung, Taiwan Republic of China 40447	Martin M-T Fuh MD, DMSci
Central IRB - United States; Sites 32001, 32002, 32003, 32004, 32005, 32007, 32008, 32010, 32011, 32012, 32013, 32014, 32015, 32016, 32017, 32018, 32019, 32020, 32022, 32023, 32025, 32026, 32027, 32028, 32029, 32031, 32032, 32033, 32034, 32037, 32038, 32039, 32040, 32041, 32042, 32043, 32044, 32045, 32046, 32047, 32049, 32050, 32052, 32053, , 32056, 32057, 32059, 32060, 32061, 32062, 32063, 32064, 32065, 32066, 32067, 32068, 32069, 32070, 32071, 32072, 32074, 32075,	Quorum Review IRB 1501 Fourth Ave Suite 800 Seattle, Washington United States 98101	Stephen J. Rosenfeld, MD, MBA

Study center number	Name of the IRB	Chairperson
32076, 32079, 32080, 32081, 32084, 32085, 32086, 32087, 32088, 32089, 32090, 32092, 32093, 32094, 32096, 32097, 32098, 32101, 32102, 32104, 32105, 32106, 32107, 32108, 32109, 32110, 32113, 32114, 32115, 32116, 32117, 32119, 32121		
LEC - United States; Site 32054	Quorum Review IRB 1501 Fourth Ave, Suite 800 Seattle, WA 98101	Stephen Rosenfeld
LEC - United States; Sites 32009, 32035	WIRB South Hill Business and Technology Center 1019 39th Ave SE Puyallup, Washington United States 98374	R. Bert Wilkins, JD, MHA, CIP
LEC - United States; Site 32021	Creighton University IRB 2500 California Plaza Omaha, Nebraska United States 68178	Mary Kunes-Connell, RN, PhD
LEC - United States; Site 32024	Mercy Medical Center - DSM 1111 6th Avenue Des Moines, Iowa United States 50314	Matthew Andres, DO
LEC - United States; Sites 32099, 32055, 32078	BRANY IRB 10 Union Square East - 5J Suite 210 Lake Success, New York United States 11041	Co-chairs: Mark J. Sinnett, PharmD and Ira Leviton, MD
